# In Situ Gel with Silver Nanoparticles Prepared Using *Agrimonia eupatoria* L. Shows Antibacterial Activity

**DOI:** 10.3390/life13020573

**Published:** 2023-02-17

**Authors:** Ľudmila Balážová, Tomáš Wolaschka, Simona Rohaľová, Nina Daneu, Martin Stahorský, Aneta Salayová, Ľudmila Tkáčiková, Jarmila Eftimová

**Affiliations:** 1 Department of Pharmaceutical Technology, Pharmacognosy and Botany, University of Veterinary Medicine and Pharmacy in Košice, 041 81 Kosice, Slovakia; 2 Department of Pharmaceutical Technology, Faculty of Pharmacy, Masaryk University, 601 77 Brno, Czech Republic; 3 Advanced Materials Department, Jozef Stefan Institute, SI-1000 Ljubljana, Slovenia; 4 Department of Mechanochemistry, Institute of Geotechnics, Slovak Academy of Sciences, 040 01 Kosice, Slovakia; 5 Department of Chemistry, Biochemistry and Biophysics, Institute of Pharmaceutical Chemistry, University of Veterinary Medicine and Pharmacy in Košice, 041 81 Kosice, Slovakia; 6 Department of Microbiology and Immunology, University of Veterinary Medicine and Pharmacy in Košice, 041 81 Kosice, Slovakia

**Keywords:** nanoparticles, silver, *Agrimonia eupatoria*, in situ gel

## Abstract

Silver nanoparticles (Ag NPs) with antibacterial activity can be prepared in different ways. In our case, we used ecological green synthesis with *Agrimonia eupatoria* L. The plant extract was used with Ag NPs for the first time to prepare termosensitive in situ gels (ISGs). Such gels are used to heal human or animal skin and mucous membranes, as they can change from a liquid to solid state after application. Ag NPs were characterized with various techniques (FTIR, TEM, size distribution, zeta potential) and their antibacterial activity was tested against *Staphylococcus aureus* and *Escherichia coli*. In accordance with the TEM data, we prepared monodispersed spherical Ag NPs with an average size of about 20 nm. Organic active compounds from *Agrimonia eupatoria* L. were found on their surfaces using FTIR spectroscopy. Surprisingly, only the in situ gel with Ag NPs showed antibacterial activity against *Escherichia coli*, while Ag NPs alone did not. Ag NPs prepared via green synthesis using plants with medicinal properties and incorporated into ISGs have great potential for wound healing due to the antibacterial activity of Ag NPs and the dermatological activity of organic substances from plants.

## 1. Introduction

In situ gels (ISGs) are novel gel-based dosage forms and delivery systems [1] (Cui, 2023). ISGs are liquid dosage forms that create a gel at the application site under the influence of environmental conditions [2]. The main advantage of ISGs is that sol–gel transition and gel formation are reversible and do not require a potentially toxic cross-linking agent [3]. ISGs are prepared from polymer excipients called smart polymers that can alter their physicochemical properties in response to environmental changes. Their fluidity before application and subsequent gel formation capability make them great candidates for oromucosal delivery in the treatment of oral diseases. Further advantages of ISGs include their capacity to be administered into or onto various types of tissues (both healthy and affected); their mucoadhesivity; their prolonged retention time; their capacity for sustained or controlled release; and patient comfort [4].

In this study, an termosensitive in situ gel with nanoparticles (NPs) was improved. NPs have different properties compared to bulk particles, mainly due to their surface-to-size ratio. Synthesis of NPs can be undertaken through physical, chemical, or biological methods [5,6]. A more ecological biological synthesis method for NPs involves mediation by various biological substances (bacteria, micromycetes, yeasts, viruses, algae, plants, and plant extracts [7,8,9,10,11]. The secondary metabolites of plants are able to reduce silver, which is the precursor of silver nanoparticles. In addition, the organic matrix can bind to the NPs’ surface and is responsible for stabilization and other effects. In our case, we chose a plant (*Agrimonia eupatoria* L.) that is used for the treatment of oral cavity disorders for the preparation of Ag NPs and the subsequent in situ gel.

*Agrimonia eupatoria* L. (also called church steeples, stickwort, common agrimony, and liverwort) is a perennial herb that has been used in folk medicine for its beneficial effects since the time of Ancient Greece [12]. It has been used for gastrointestinal disorders, diarrhea, cholecystitis, pneumonia, hepatopathy, pyelonephritis, cystitis, bleeding disorders, skin defects, and oral mucosal inflammatory diseases [13,14]. The results of published studies have confirmed its anti-inflammatory, astringent, diuretic, antimicrobial, antiviral, antioxidant, neuroprotective, and hepatoprotective effects, as well as many others [15]. It contains a number of secondary metabolites (polyphenols, tannins, flavonoids) with the ability to reduce silver to nanoscale silver.

In this paper, we describe the preparation of silver nanoparticles from the plant extract of *Agrimonia eupatoria* L. and their characterization. This was also a preliminary study in which silver nanoparticles were used for the preparation of ISGs for the first time, and we also measured their antibacterial activity.

## 2. Materials and Methods

### 2.1. Chermicals

Pluronic F 127 and Methylcellulose Ph. Eur. were obtained from Sigma-Aldrich (Saint-Louis, MO, USA). Noaveon AA-1 was kindly gifted by Lubrizol (Wickliffe, OH, USA). AgNO_3_ (99.9%) was obtained from Mikrochem (Pezinok, Slovakia). The HPLC standards rutin trihydrate, quercetin, and gallic, caffeic, p-coumaric, o-coumaric, rosmarinic, and trans-cinnamic acids were purchased from Sigma–Aldrich (St. Louis, MO, USA). HPLC solvents were purchased from Fischer (Fisher Scientific UK Ltd., Loughborough, UK).

### 2.2. Plant Material Preparation and Characterization

The plant *Agrimonia eupatoria* L. was collected from the nature environment in Malá Tŕňa, Slovakia. The exact identification of the collected plant was undertaken by the Department of Pharmaceutical Technology, Pharmacognosy and Botany, University of Veterinary Medicine and Pharmacy, Košice, Slovakia. After collection, aerial parts in the flowering stage were washed with distilled water to remove all stones, dust, and undesirable debris. The clean plant was dried to a constant weight (Concept SO 1030, Jindřich Valenta—Concept, Chocen, Czech Republic). The drying temperature was 40 °C, at which level degradation of metabolites does not occur. To increase extraction yield, the dried plant was ground with an electric grinder (Sencor SCG 1050WH, Yiwu, China). The plant was stored in dry and dark conditions to prevent the destruction of the metabolites. The extract was prepared from 1 g of ground material in 100 mL of distilled water through sonication (KRAINTEK 18, Kraintek s.r.o., Podhájska, Slovakia) for 1 h. Subsequently, we filtered the contents of each Erlenmeyer flask through filter paper. The extract was prepared immediately before further processing.

#### 2.2.1. Phytochemical Characterization

Phytochemical characterization and characterization of the antioxidant activities of the *Agrimonia eupatoria* L. extracts were undertaken spectrophotometrically and with HPLC analysis. The total amounts of polyphenols present in the individual extracts were determined via the colorimetric method using Folin-Ciocalteau reagent and calculated using the equation for the gallic acid calibration curve in mg GAE/Ll sample (galic acid equivalent) [16]. Determination of the total flavonoid content was undertaken spectrophotometrically at a wavelength of 415 nm (Beckman DU 530 UV/VIS, Delta Electronics, Pudong Shanghai, China). The flavonoid content was calculated as quercetin using the equation for the quercetin calibration curve in mg QE/L sample (quercetin equivalent) [17]. The concentration of total phenolic acid was measured spectrophotometrically and calculated as the chlorogenic acid equivalent (mg CGAE/L) [18]. The antioxidant activity was determined using the DPPH free radical scavenging method (2,2-diphenyl-1-picrylhydrazyl radicals) [19].

#### 2.2.2. HPLC Analysis

HPLC-DAD analysis was performed with an UHPLC Dionex UltiMate 3000 RS system (Thermo Fisher Scientific, Waltham, MA, USA) equipped with an autosampler, binary pump, thermostat, and diode array detector (DAD). Samples of *Agrimonia eupatoria* L. prepared in HPLC-grade water were filtered through a PVDF syringe filter (0.25 µm) prior to injection. Stock solutions of the standards chlorogenic acid, gallic acid, caffeic acid, p-coumaric acid, o-coumaric acid, rutin trihydrate, rosmarinic acid, and cinnamic acid were prepared in methanol at a concentration of 1000 mg·mL^−1^ and then standard solutions were prepared in concentrations ranging from 0.1 to 20 mg·L^−1^ in mobile phase A. For the stationary phase, a Kinetex 2.6 µm F5 column (100 Å, LC Column 150 × 4.6 mm) with a PFP pre-column SecurityGuard from Phenomenex (Torrance, CA, USA) was used. Samples were eluted using a mobile phase containing 0.1% formic acid in water (solvent A) and methanol (solvent B), with the elution gradient as follows: 0 min 20% B, 20 min 75% B, 29 min 90% B, 29–34 min 20% B under isocratic conditions; the overall elution time was 34 min at a flow rate of 0.8 mL·min^−1^. The column was thermostatted and the temperature adjusted to 30 °C. The injection volume was 5 µL. Detection was carried out using a DAD detector and evaluated at different wavelengths according to the absorption maxima of the analyzed compounds, which were 280 nm (gallic and cinnamic acid), 309 nm (p-coumaric acid), 328 nm (chlorogenic acid, o-coumaric acid, rosmarinic acid, and caffeic acid), and 373 nm (quercetin). The identification of phenolic compounds was carried out by comparing their retention times with the corresponding standards. Chromeleon 7.2 software was used for instrument control and evaluation of results.

### 2.3. Green Synthesis of Silver Nanoparticles

The synthesis of silver nanoparticles was undertaken with 1 mM aqueous solution of silver nitrate (AgNO_3_) and water extract of *Agrimonia eupatoria* L. prepared as described above. The substances were mixed in a ratio of 9:1. First, 1 mM AgNO_3_ was heated in a water bath and mixed with a stirrer. When the temperature was between 75 and 80 °C, an adequate amount of plant extract was added dropwise. The solution was heated for 30 min at 75–80 °C and then cooled to room temperature and placed in the refrigerator (4–8 °C). The preparation of silver nanoparticles was undertaken under dark conditions to minimize the photochemical degradation of AgNO_3_. The Ag NPs were prepared freshly immediately before use.

### 2.4. Characterization of Silver Nanoparticles

#### 2.4.1. Spectrophotometric Analysis

The process of silver nanoparticle formation was monitored in situ every minute using a Cary 60 UV–Vis spectrophotometer (Agilent Technologies, Victoria, Australia) with a heated Peltier block (Single Cell Peltier accessory, Agilent Technologies, Victoria, Australia), and the spectra were recorded in the range from 300 to 700 nm in a quartz cuvette (10 mm).

#### 2.4.2. Transmission Electron Microscopy

Microstructural analyses of the silver nanoparticles after plant extract-assisted synthesis were performed using transmission electron microscopy (TEM). For TEM analyses, the suspension containing the nanoparticles was sonicated for a few minutes, applied onto a lacey carbon copper grid, and dried. Prior to the TEM analyses, the grid was coated with a thin layer of carbon to prevent charging under the electron beam.

#### 2.4.3. Fourier Transform Infrared Spectroscopy (FTIR)

Fourier transform infrared (FTIR) analysis was performed with a Tensor 29 (Bruker, Berlin, Germany) with an ATR technique. FTIR spectra of the aqueous extract of *Agrimonia eupatoria* L. before and after biosynthesis of Ag NPs were recorded in the region of 4000–650 cm^−1^. Additionally, the FTIR spectra of the Ag NPs were obtained. The Ag NPs were washed to remove all of the extract and keep only chemicals from *Agrimonia eupatoria* L. connected to the Ag NPs. The reaction mixture was centrifuged for 10 min at 15,000 rpm using an Eppendorf Centrifuge 5430 (Eppendorf, Hamburg, Germany). The supernatant was removed. The pellet was resuspended in distilled water and the process was repeated five times.

#### 2.4.4. Size Distribution

The particle size distribution for the nanosuspensions produced was determined in water using a Mastersizer^®^ 3000 with a Hydro MV wet dispersion attachment (Malvern Panalytical Ltd., Malvern, UK) and a refractive index of 1.33 for water. Small amounts of nanosuspension were placed directly into the Hydro MV unit with a constant stirrer speed of 3000 rpm in small increments until an obscuration rate of ~3% was achieved. Each sample was subjected to pulsed sonication at 50% in 10 s intervals with the same pause length. The particle size distributions were calculated in Mastersizer 3000 software (version 3.0) using Mie theory. The refractive index (RI) and the absorption index (AI) were set for Ag particles (RI: 0.135; AI: 3.99; source: Malvern database for silver). The RI and AI for both the in situ gel and the in situ gel loaded with Ag NPs were set as 1.47 and 0.01, respectively (source: Malvern database for cellulose). Three repetitions were analyzed for each sample, and the results reported are the averages of all scans for the volume median diameters d(0.1), d(0.5), and d(0.9).

#### 2.4.5. Zeta Potential

The zeta potential (ZP) was determined using a Zetasizer Nano ZS (Malvern, Great Britain, UK) by applying the Smoluchowski equation built into Malvern Zetasizer software. The ZP was measured in demineralized water with the addition of 10 mM NaCl to maintain a minimum level of conductivity in the medium. The measurements were repeated at least three times for each sample in the time sequence: on the day of preparation and the 2nd, 3rd, 7th, and 14th days after preparation. The zeta potential measurement was performed at room temperature (25 °C) when the in situ gel was in a liquid state. The samples were kept in a refrigerator (4 °C).

### 2.5. Preparation of Sols

To remove powder aggregates, methylcellulose (MC) and Noveon AA-1 (NA1) were sifted using a 0.8 mm sifter (Preciselekt s.r.o., Dolní Loučky, Czech Republic). MC was dispersed in two thirds of the calculated volume of distilled water heated at 80 °C. The weighted amount was slowly added to the beaker with agitated water at 450 rpm and stirred for another 5 min (Witeg Labortechnik, Wertheim, Germany). Then, NA1 was added in the same manner and stirred for 5 min. The remaining third of the distilled water, cooled at 4 °C, was added and stirred, and then the dispersion was placed in the refrigerator for at least 20 min. Pluronic F 127 (P127) was dispersed using the cool method described by [20]. It was slowly added to the cooled dispersion under continuously stirring at 550 rpm. The stirring was continued for another 10 min. The sol was placed in the refrigerator for at least 20 h.

### 2.6. Preparation of Sols Loaded with Nanoparticles

Sols loaded with Ag NPs were prepared in the same way as mentioned above but, instead of distilled water, a suspension with Ag NPs was used.

### 2.7. Stability of Nanoparticles and In Situ Gels Loaded with Ag NPs

The stability of Ag NPs was evaluated spectrophotometrically after storing the samples under various environmental conditions; specifically, at 4–8 °C (Xy) and at room temperature in an environment with light exposure (Yz). Samples of Ag NPs, ISGs loaded with Ag NPs, and ISGs without Ag NPs were compared. The absorbance of the samples was determined after the preparation (0 h) and then after 24 h, 48 h, 7 days, and 14 days. Before measurement, samples were diluted with distilled water in a ratio of 1:10. Spectra were recorded using a Cary 60 spectrophotometer (Agilent Technologies, Victoria, Australia) in the range from 300 to 600 nm in a quartz cuvette (10 mm).

### 2.8. Antibacterial Activity

Antibacterial properties were tested using the plate agar diffusion method [21]. The activities of the silver nanoparticles, agrimony extract, and 1 mM AgNO_3_ were tested against Gram-positive and Gram-negative bacterial strains (*Staphylococcus aureus* CCM 4223 (SA) and *Escherichia coli* CCM 3988 (EC)). Distilled water was used as a negative control and gentamicin sulfate (Biosera, Nuaille, France) with a concentration 50 μg/mL as a positive control. The plates were incubated at 37 °C for 20 h. After incubation, the sizes of the inhibition zones were evaluated using ImageJ 1.48 software (NIH Image, Bethesda, MD, USA). The antibacterial effects of the samples were recalculated according to Equation (1):

% RIZD = [(IZ_Sample_ − IZ_Negative control_)/IZD_gentamicin_] × 100 (1)

where RIZD expresses the percentage of the average relative inhibition zone and IZD is the diameter of the inhibition zone in mm.

### 2.9. Statistical Analysis

GraphPad Prism 5 (GraphPad, San Diego, CA, USA) was used for statistical analysis. The results were expressed as means ± SEM and values of *p* < 0.05 (*), *p* < 0.01 (**), and *p* < 0.001 (***) were considered significant.

## 3. Results and Discussion

### 3.1. Phytochemical Characterization

**Figure 1:**
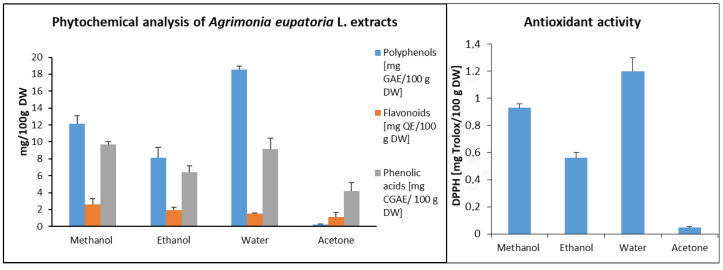
Phytochemical analysis and antioxidant activity of different extracts of *Agrimonia eupatoria* L. GAE—galic acid equivalent, QE—quercetine equivalent, CGAE—chlorogenic acid equivalent, DW—dry weight.

Since the phytochemical composition of the extract strongly influences the bioreduction of silver ions, different methods of plant characterization were employed to provide a comprehensive view of the mechanism of nanoparticle biosynthesis. The phytochemical characterization of different extracts of *Agrimonia eupatoria* L. was focused on the determination of the secondary metabolites responsible for the synthesis of nanoparticles. The biosynthesis of silver nanoparticles is based on the reduction of silver ions from AgNO_3_ to neutral silver. With the correct synthesis conditions, the silver created will be nanosized. Plant extracts contain many primary, as well as secondary, metabolites with reducing abilities. Proteins and saccharides from the group of primary metabolites can be mentioned. The secondary metabolites with reducing abilities are flavonoids, phenolic acids, tannins, vitamins, essential oils, and so on. The results showed that all the *Agrimonia eupatoria* L. extracts prepared with the different solvents (water, methanol, ethanol, acetone) contained polyphenols [22]. The highest concentration of total polyphenols was in the water extracts (18.54 ± 0.4 mg/100 g of dry weight (DW)) (Figure 1). The lowest concentration was in the extracts prepared with acetone (1.5 ± 0.1 mg/100 g DW). From the results obtained, we can conclude that the polyphenols of *Agrimonia eupatoria* L. have a generally polar character. Therefore, they can dissolve better in polar solvents, such as water. The polarity of the polyphenols was responsible for the larger amounts of polar groups, such as –OH, –COOH, and =O, as well as the bond with polar mono- and disaccharides. Tannins from *Agrimonia eupatoria* L. were identified only in the aqueous extracts [14,23,24]. Other scientists have also reported that phenolic acids were mostly obtained from aqueous extracts [24,25].

Polyphenols are a large group of secondary metabolites consisting of tannins, flavonoids, phenolic acids, anthocyanins, and coumarins. This means that the concentration of measured flavonoids should have been lower. Concretely, it was more than ten times lower than the concentration of polyphenols. The total flavonoid contents in the edible extracts decreased in the following order: methanolic (2.58 ± 0.7 mg QE/100 g DW), ethanolic (1.96 ± 0.3 mg QE/100 g DW), aqueous (1.51 ± 0.1 mg QE/100 g DW), and acetone (1.13 ± 0.5 mg QE/100 g DW). Similar results—i.e., that the content of flavonoids was more than ten times lower than the content of all polyphenols—were obtained by Kubinová [26]. Our results concerning polyphenols are supported by other authors who found different substances in *Agrimonia eupatoria* L. From a group of flavonoids, researchers have detected apigenin derivatives [25], such as isovitexin (apigenin 6-C-glucoside) [26]; luteolin derivatives [27]; quercetine derivatives, such as rutin (quercetine 3-O-rhamnoglucoside) [24,28]; and kaempferol derivatives, such as astragalin (kaempferol 3-O-glucoside) [29]. Condensed tannins, such as agrimoniin [23] and hydrolyzable tannin pedunculagin [27], were isolated. We determined the highest total phenolic acid contents (9.71 ± 0.3 mg CGAE/100 g DW) in the methanol extract. We measured slightly lower values (9.15 ± 1.3 mg CGAE/100 g DW) in the water extract of *Agrimonia eupatoria* L. The acetone extract showed the lowest total phenolic acid contents (4.18 ± 1.0 mg CGAE/100 g DW).

Antioxidant activity was measured using the DPPH reaction (Figure 1). Increasing values for the equivalent concentration of Trolox were found in the following order: acetone (0.046 ± 0.008 mg Trolox/100 g DW), ethanol (0.56 ± 0.04 mg Trolox/100 g DW), and methanol (0.93 ± 0.03 mg Trolox/100 g DW). The highest activity was determined in water (1.2 ± 0.1 mg Trolox/100 g DW). This was confirmed by the total amount of polyphenols involved in antioxidant activity. Although the highest concentrations of phenolic acids and flavonoids were measured in the methanolic extract, the strong antioxidant effect was measured in the aqueous extract. The highest concentration of polyphenols was found in the aqueous extract, and, in our case, polyphenols were more responsible for antioxidant activity than flavonoids or phenolic acids. Antioxidant activity also depends on the structures of polyphenols and other substances dissolved in water.

Based on the findings that the largest amounts of polyphenols were in the water extract and the strongest antioxidant activity was also measured in water, we selected this sample for the green synthesis of silver nanoparticles. The other motivation for the selection of water as a solvent was that, in contrast to methanol or acetone, it is not toxic.

We next describe the HPLC-DAD analysis of the selected polyphenols in the aqueous extract that was undertaken in order to obtain a qualitative/quantitative profile of the tested extract. Table 1 summarizes the spectral and chromatographic characteristics of the investigated reference standards. Among them, the chromatogram revealed the presence of seven of the evaluated phenolic compounds. The main phenolic compound detected was rutin, showing a concentration of 6.56 mg·L^−1^. In addition, among the phenolic acids contained in *A. eupatoria* L., *p*-coumaric acid, *o*-coumaric acid, caffeic acid, and chlorogenic acid (in order of decreasing quantity) were determined. Moreover, previous studies indicated the presence of other phenolic constituents, mainly apigenin, kaempferol, and quercetin derivatives, as well as catechin and oligomeric proantocyanidins [25]. In contrast to our study, rosmarinic acid has previously been found to be the dominant compound in the methanolic extract of *A. eupatoria* L. In addition to rosmarinic acid, rutin, apigenin, kaempferol, and quercetin (in order of decreasing quantity) were also determined [30]. In our aqueous extract, rosmarinic acid and quercetin were not detected, and the HPLC chromatogram revealed only small amounts of gallic and cinnamic acid. According Huzio et al. [31], the concentrations of gallic and cinnamic acid were 8.0 ± 0.3 and 225.2 ± 4.1 mg/100 g, respectively.

### 3.2. Green Synthesis

**Figure 2:**
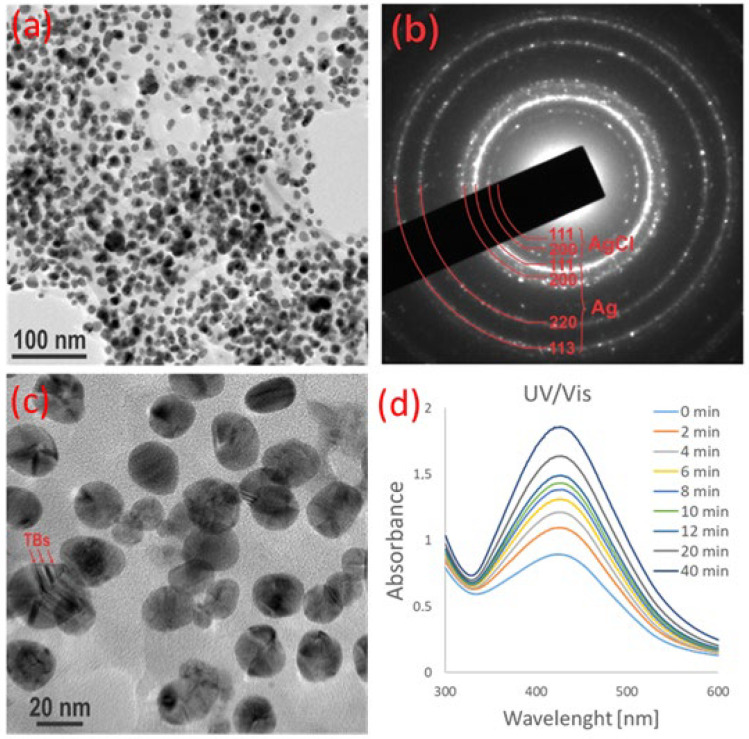
TEM and UV–Vis analyses of Ag NPs prepared with *Agrimonia eupatoria* extract. (**a**) A micrograph at lower magnification revealed that the nanoparticles were embedded in an organic matrix and had a uniform size distribution. (**b**) The SAED pattern contained weak reflections from AgCl in addition to silver, which was the main phase in the sample. (**c**) A higher magnification image of the Ag NP showed that the NPs were faceted and sometimes contained twin boundaries (TBs, marked by arrows). (**d**) UV–Vis spectra for representative times showing synthesis of Ag NPs.

Silver nanoparticles were prepared using a fast, economically advantageous, and ecologically friendly method of biosynthesis. The nanomaterial synthesis was based on the reduction of silver ions (Ag^+^, from AgNO_3_) to Ag^0^. The chemicals present in the plant extract were responsible for the reduction of silver ions to nanosilver. The synthesis was carried out at a temperature of 80 °C because the same temperature was used for the preparation of the ISGs. During the synthesis, we noticed a visual change caused by the Ag NPs. The nanoparticles changed the color of the solution from the initial light brown-yellow to dark brown. Similar observations have been reported for *Agrimonia pilosa* Ledeb. [32] and other plants [5,7]. Visual changes were monitored spectrophotometrically, which is the main tool used to analyze the formation of silver and gold nanoparticles. Increases in absorbance occurred at almost all wavelengths but especially around 425 nm (Figure 2d). In the case of *Agrimonia pilosa* Ledeb., the Ag NPs synthesized with the plant extracts were found to have a different spectrum peak; however, it was mainly observed from 420 to 470 nm. Maximum optical absorptions for previous prepared NPs were found at 445 nm [32] for *Thymus serpyllum* L. and 469 nm [33] and 436 nm for *Sambucus nigra* L. [34]. The UV–Vis spectra of silver nanoparticles prepared via green synthesis depend on the surface plasmon resonance (SPR). The shape, size, aggregation, and surrounding dielectric medium of nanoparticles are responsible for the value of the SPR [35]. It has been found that the size of nanoparticles and the absorption peak are related to each other. A peak near smaller wavelengths indicates a smaller NP size; on the other hand, higher wavelengths indicate larger nanoparticles [36]. The small size of the nanoparticles was confirmed using TEM and size distribution analyses.

### 3.3. Characterization of Nanoparticles

#### 3.3.1. Transmission Electron Microscopy

Low-magnification micrographs of the silver nanoparticles after 7 days of synthesis are shown in Figure 2a. They reveal that the nanoparticles had an isometric morphology and quite uniform particle size distribution. The nanoparticles were attached to the organic matrix, as is typical for a plant-assisted biosynthesis approach. Measurement of approximately 60 particles revealed that the average diameter of the nanoparticles was 20.2 ± 4.6 nm. The crystalline structure of the nanoparticles was determined using selected electron diffraction (SAED) and demonstrated diffraction rings (Figure 2b). The measurement of the d-values revealed that the main phase in the sample was silver and, in addition, a low amount of silver chloride (AgCl) was detected (the two weaker innermost rings with the highest d-values). A higher magnification image of the silver nanoparticles (Figure 2c) indicated that they were faceted and contained planar defects, most likely twin boundaries, as a consequence of the presence of AgCl in the sample. The fraction of AgCl was estimated to be less than 5%. It is likely that AgCl NPs contributed to the overall anti-inflammatory activity of the sample [37]. Synthesis of AG NPs and AgCl nanoparticles using plants has also been demonstrated by other authors [38,39].

#### 3.3.2. FTIR Analysis

**Figure 3:**
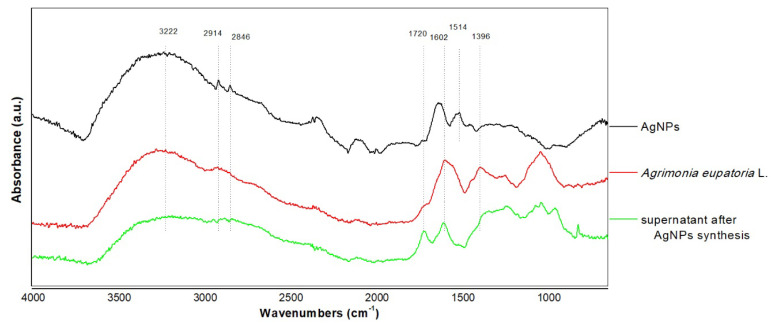
FTIR analysis of *Agrimonia eupatoria* L. before and after synthesis of Ag NPs and spectrum of Ag NPs.

FTIR analysis was performed to characterize the phytochemicals involved in the bioreduction of Ag^+^ or the capping of the formed nanoparticles prepared from aqueous extract of *Agrimonia eupatoria* L. For this purpose, the FTIR spectra of the dried extract of *Agrimonia eupatoria* L., the nanoparticles separated by centrifugation, and the dried supernatant after synthesis of Ag NPs, respectively, were measured (Figure 3). These FTIR spectra provided information about the mechanism of nanoparticle formation using the metabolites contained in the plant extract. The FTIR spectrum of the Ag NPs showed many absorption bands, indicating strong interaction between and capping of biomolecules on the nanoparticles’ surface. Several bands characteristic of the crude extract of *Agrimonia eupatoria* L. were also found in the spectrum of the Ag NPs, as summarized in Table 2. Based on the absorption bands, the presence of characteristic chemical structures, such as alcohol, phenol, carboxylic acid, amine, and carbonyl groups, could be estimated. The strong visible band at 3222 cm^−^^1^ was assigned to O–H (alcohol, flavonoids, and phenolic compounds). The two sharp peaks visible at 2914 and 2846 cm^−^^1^ were attributed to the C–H bond stretching vibrations of methyl groups. These vibrations were almost similar and were found in all three samples. A difference was found at about 1720 cm^−^^1^, where the increase in the absorption band in the supernatant spectra may have indicated the formation of the carbonyl group during the formation of Ag NPs. The C=C aromatic ring stretching vibration bands manifested at 1637, 1514, and 1456 cm^−^^1^ in Ag NPs, but in the agrimony extract and supernatant, they were shifted to about 1604 cm^−^^1^. Furthermore, the OH bending vibration of the phenol group was detectable at 1396 and 1371 cm^−^^1^ in the *Agrimonia eupatoria* L. extract and supernatant and was weakened after bioreduction in the Ag NPs’ spectra. The region from 1246 to 1039 cm^−^^1^, presumably corresponding to the stretching vibrations of the −C−O−C ether group or the C–O alcohol bond and C–C stretching skeletal vibrations, disappeared in the Ag NPs.

The water-soluble compounds of the extract were responsible for the silver ion reduction and bonded to the surfaces of the nanoparticles through various function groups to stabilize them and avoid future aggregation. The mechanism underlying the reduction and stabilization of silver nanoparticles has already been studied using flavonoids, rosmarinic acid, and luteolin-7-glucoside [40], as well as phenolic acids; namely, sinapic acid [41], caffeic acid [42], and gallic acid [43]. Briefly, the hydroxyl group undergoes oxidation to the corresponding quinone structure, leading to a simultaneous reduction of Ag^+^ to Ag^0^. Similarly, in our case, according to the phytochemical and FTIR analysis, the phenolic compounds seemed to be responsible for the bioreduction and stabilization by covering the prepared Ag NPs.

The phytochemical composition of *Agrimonia eupatoria* L. shows that it contains a large number of pharmacologically active compounds. According to previous analyses, green herbs with flowers contain more than 2% phenolic acid [24,44], around 2% tannins [24], 1.2–1.4% flavonoids [44,45], volatile oils [46], triterpenoids [47], acids, vitamins, and other constituents. The TEM analysis showed the biomacromolecular “shell” around the biosynthesized Ag NPs.

### 3.4. Preparation of In Situ Gel

**Figure 4:**
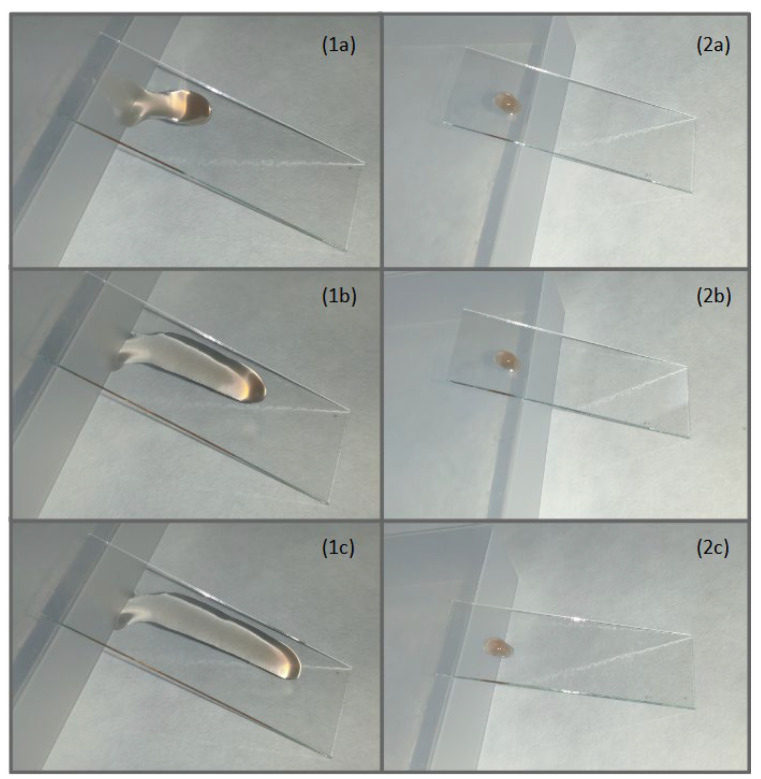
The gelation process for in situ gel loaded with silver nanoparticles. (1) Liquid sol at laboratory temperature after (**a**) 0, (**b**) 2, and (**c**) 4 s. (2) Gel heated at physiological temperature after (**a**) 0, (**b**) 2, and (**c**) 4 s.

In this study, we used Pluronic F 127 (P127), Methylcellulose Ph. Eur. (MC), and Noaveon AA-1 (NA1) for the preparation of ISGs loaded with silver nanoparticles. P127 is a water-soluble, non-ionic, triblock copolymer that consists of hydrophobic polyoxypropylene and two hydrophilic units of polyoxyethylene. It is a thermosensitive polymer and, when the critical micelle temperature is exceeded, tightly packed micelles are formed, visually detectable as a sol–gel transition [48]. MC is a non-ionic, water-soluble derivative of cellulose and has the property of being thermosensitive; however, the temperature for the transition from sol to gel is approximately 60 °C when used in concentrations effective for ISGs [49]. MC also acts as a viscosity-modifying agent with mucoadhesive properties [50]. NA1 is a pH-sensitive anionic polymer of acrylic acid; although insoluble in water, it has a high swelling capacity at pH 4 to 6. Gel formation takes place when intramolecular repulsion occurs after ionization of the polymer by the neutralizer. NA1 is highly mucoadhesive due to its carboxylic acid groups, which bind to mucosal surfaces through a hydrogen bond interaction [51]. Sol consisting of Pluronic F 127 (15%), Methylcellulose Ph.Eur. (0.5%), and Noveon AA1 (0.125%) was prepared. The loading of Ag NPs onto these polymers could be beneficial since they tend to aggregate, and the polymeric network can prevent this from happening [52]. The prepared in situ gel appeared glassy without turbidity or opalescence, and its pH was 4.97 ± 0.045. The critical temperature of the sol–gel transition was more than 30 °C (Figure 4), meaning that this model is suitable for application on mucosa. After application of the solution to the mucous membrane, it changed from a liquid to a solid state. Therefore, it could remain fixed to the mucous membrane for a longer time, helping to protect the mucous membrane from external factors and prolong the dissolution of the medicine incorporated inside. In our case, we employed Ag NPs with antibacterial activity and secondary metabolites from *Agrimonia eupatoria* L. with astringent, antioxidant, and anti-inflammatory activities. Dispersion of polymers in Ag NP solutions was possible without complications. Incorporation of Ag NPs into ISGs did not cause any visible aggregation of the nanoparticles. We found that it was possible to prepare an in situ gel in combination with biosynthesized silver nanoparticles while maintaining the original technological function and, in addition, improving the biological function.

### 3.5. Stability of Ag NPs and Ag NPs in Gel

#### 3.5.1. Size Distribution

**Figure 5:**
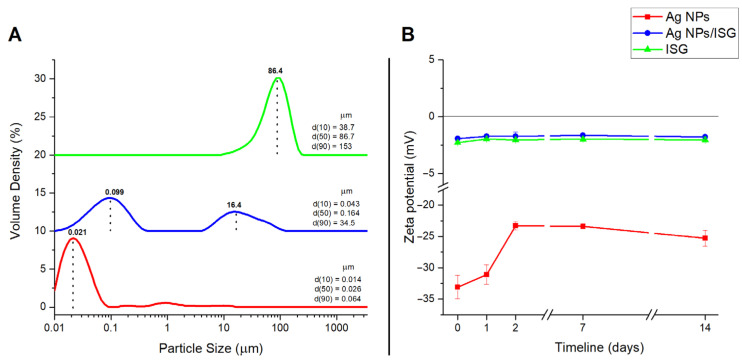
(**A**) The particle size distributions and (**B**) zeta potential of Ag NPs (red), the ISG loaded with Ag NPs (blue), and the ISG (green).

The particle size distributions of the freshly prepared Ag NPs, as well as the in situ gel loaded with Ag NPs and thermosensitive in situ gel, are shown in Figure 5A. The volume median diameters d(0.1), d(0.5), and d(0.9) were determined as identifiers of the particle size distribution for each sample. The Ag NPs showed a unimodal distribution at the nanoscale, starting even below the detection limit of the device (below 10 nm), with a small intensity of agglomerate peaks in the microregion (0.15–16 µm). The average volume median d(50) value was equal to 26 nm, and the width of the particle distribution ranged from 14 nm d(10) to 64 nm d(90). The value of d(50) was consistent with the analysis of the Ag NPs using TEM (20.2 ± 4.6 nm), but higher values for d(50) were expected for the overall volume distribution of the particles themselves, as well as for the agglomerates. These results, which rather corresponded to individual particles, may have been caused by the very intensive mixing and sonication conditions during the measurements. The in situ gel showed a broad unimodal distribution in the microregion, with an average value for the volume median d(50) of 86.7 µm.

Measurement of the two-component system of the in situ gel loaded with Ag NPs using dynamic light scattering (DLS) was carried out assuming the encapsulation of Ag NPs in the gel under the conditions of the bulkier polymer gel. A bimodal distribution for the particles with a d(50) of 0.164 µm was achieved. The main peak was located in the nanoregion, with the highest intensity corresponding to 99 nm. Presumably, this determined the size distribution of the finest Ag NPs. When Ag NPs were introduced into the ISG, the size of the Ag NPs changed from approximately 21 nm to 99 nm. The ISGs changed the charge on the surfaces of the NPs (determined by the zeta potential), which could have led to the aggregation of nanoparticles. Another possibility is that the NPs were coated with larger gel components, resulting in an increased signal. The second, less intense peak was located in the microregion, and the highest intensity corresponded to 16.4 µm, determining the size distribution of the mainly agglomerated particles with a predominance of in situ gel. Thus, the size distribution of Ag NPs increased directly with the addition of the in situ gel. According to the ATB activity (mentioned later), the size of the Ag NPs in ISGs and the agglomeration and coagulation status have positive effects.

#### 3.5.2. Zeta Potential

The zeta potential (ZP) refers to the electrokinetic potential in colloidal systems that acts at the interface between the surface layer of a nanoparticle and the surrounding liquid (water). The value of the zeta potential indicated the stability and dispersion of the NPs in the extract of *Agrimonia eupatoria* L. (1 mM aqueous solution, pH 4.50). The introduction of Ag NPs into the ISG was also investigated in terms of ZP stability to explore the potential applications (Figure 5B). The measurements were performed by adding 10 mM of NaCl solution to maintain the minimum level of conductivity in the medium, with a resulting pH of 6.30 as a function of time (day of preparation and 2nd, 3rd, 7th, and 14th days after preparation).

The values measured for the ZP for Ag nanoparticles were negative (−33.1 ± 1.86 mV), indicating the moderate stability of the system. It was hypothesized that the negative zeta potential of the Ag NPs prepared via green synthesis was a result of the negatively charged organic matrix surrounding the nanoparticles. The organic compounds connected to the Ag NPs came from the plant extract during biosynthesis. These results were also confirmed by the FTIR analysis, which detected an organic compound on the surface of the nanoparticles. The greater advantage of preparing nanoparticles via green synthesis is that the organic material from the plant extract is fixed to the surface of the nanoparticles. These compounds affect the charge of the NPs, leading to the repulsion of the nanoparticles. Additionally, preparing nanoparticles via green synthesis prevents aggregation [53,54].

The monitoring of the sample provided information about its aging. On the second day, an insignificant decrease in the ZP of −31.1 ± 1.55 was detected. A more pronounced decrease of −23.3 ± 0.666 occurred on the third day; at the same time, this was a plateau value in relation to the further observations on the 7th (−23.4 ± 0.283) and 14th days (−25.3 ± 1.27). Negatively charged Ag NPs are sensitive to storage conditions, and such decreases in ZP values have already been observed several times [55,56].

The introduction of Ag NPs into the ISG made the ZP values rapidly decrease (−1.91 ± 0.055 mV) on the first day of preparation. The ZP value for the ISG was −2.27 ± 0.132 mV. These values indicate system instability and rapid coagulation and flocculation can be assumed. The ISG brought a change in the charge (first day—Ag NPs: −33.1 ± 1.86 mV, ISG without Ag NPs: −2.27 ± 0.132 mV, and ISG with Ag NPs: −1.91 ± 0.055 mV) and acted accordingly as a coagulant. ISG polymers can provide steric stabilization, which can contribute to decreased aggregation [57]. However, there was no significant decrease during the time course, and plateau values were observed both for the ISG itself and for the Ag NPs in the gel. It is interesting that the ISG alone always achieved absolute ZP values that were a few tenths higher than the ISG loaded with Ag NPs. This small change in the ZP could be explained by the high ratio of in situ polymers to Ag NPs. This change—in our case, the increase in ZP—was also observed after the addition of anionic surfactants to poly(methyl methacrylate) polymer solution, which was probably driven by hydrophobic-based adsorption.

#### 3.5.3. Spectroscopic Analysis

The stability of the Ag NPs was determined by detecting their presence in differently stored samples through spectrophotometric scanning of the spectrum from 300 to 600 nm. The colloidal stability of nanoparticles in living systems (defined in part by the synthesis method applied) can directly influence biological activity, an issue that cannot be overlooked, especially when these materials are designed for therapeutic uses. The absence of peaks in the ISG spectra and the presence of peaks at a wavelength of approximately 430 nm in the spectra of the ISG loaded with Ag NPs confirmed the successful incorporation of spherical Ag NPs into the gels [58].

**Figure 6:**
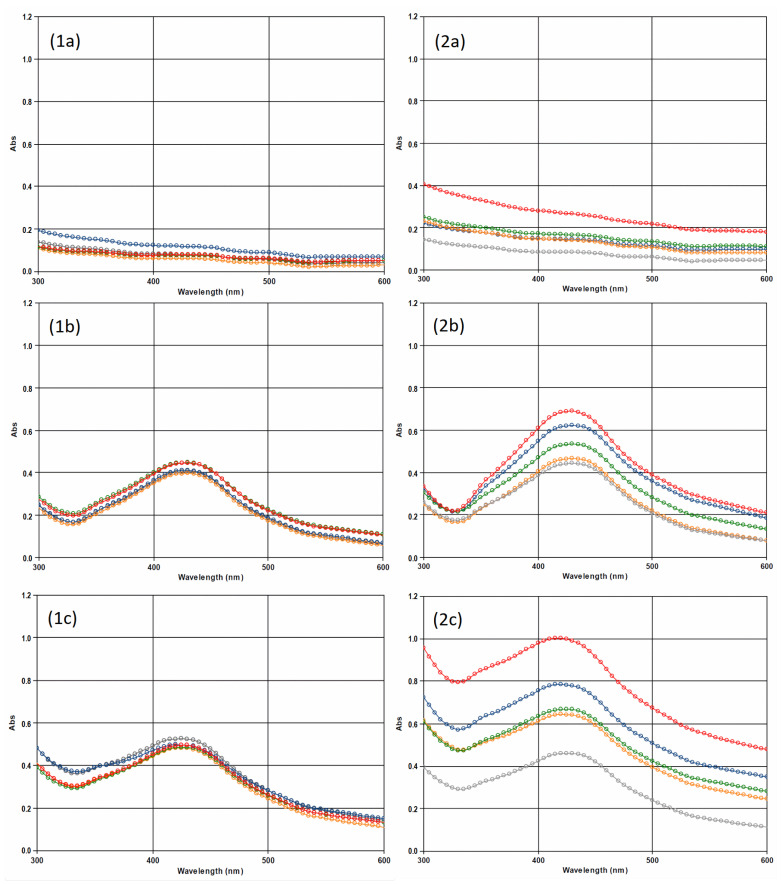
UV–Vis absorption spectra of (1) the samples stored at 4–8 °C and (2) the samples stored at laboratory temperature in an environment with light exposure: (**a**) in situ gel without Ag NPs, (**b**) Ag NPs, and (**c**) in situ gel with Ag NPs. The absorbance of the samples: 0 h (grey), 24 h (yellow), 48 h (green), 7 days (blue), 14 days (red).

In the data analysis, the absorbance of the gel was subtracted from the absorbance of the ISG with Ag NPs (Figure 6). Almost identical peaks were recorded at 430 nm for Ag NPs and ISG loaded with Ag NPs across the entire storage period (14 days) when stored at a lower temperature (0.422 ± 0.024 vs. 0.412 ± 0.024). The valley at 330 nm showed slightly higher levels of 21.1% in the ISG with Ag NPs samples (0.179 ± 0.022 vs. 0.216 ± 0.021). There were no significant deviations in the absorbance values measured with storage at low temperature.

The absorbance at 430 nm gradually increased when samples were stored at laboratory temperature in an environment with light exposure. For Ag NPs, the absorbance increased by 57.3% compared to the original (0.444 ± 0.005 compared to 0.698 ± 0.011), and for the ISG loaded with Ag NPs, it increased by 86.0% from 0.377 ± 0.020 to 0.701 ± 0.033 after 14 days.

In the valley at 330 nm, we recorded a significant difference in absorbance. While the absorbance of the Ag NPs samples increased by 30% from 0.174 ± 0.001 to 0.227 ± 0.007, for the ISG with Ag NPs samples, there was a significant increase in absorbance by 143.8% from 0.177 ± 0.015 to 0.430 ± 0.015.

The wavelengths of the peaks suggested the spherical shape of the nanoparticles [59]. These results were confirmed by the TEM analysis. The refrigeration of the samples appeared to provide an environment that ensured the stability of the Ag NPs and the ISG loaded with Ag NPs (Figure 6). We observed an increase in absorbance over time for samples stored at laboratory temperature in an environment with light exposure. Agglomeration of Ag NPs in the samples was unlikely according to results showing that the wavelength of the surface plasmon resonance peak increases when nanoparticles agglomerate and, consequently, increase in size [58]. The wavelengths of the peaks of the samples containing Ag NPs did not change or undergo minor changes.

The question remains why the increase in absorbance occurred. We know that the increase in absorbance also occurs with longer storage at a reduced temperature (internal unpublished data). As the preserved shapes of the spectra and the zeta potential results exclude agglomeration, it is most likely that further formation of Ag NPs took place, which was accelerated by the higher temperature. We will have to answer this question with further research to obtain a product with a stable level of nanoparticles suitable for further pharmaceutical processing.

### 3.6. Antibacterial Activity

**Figure 7:**
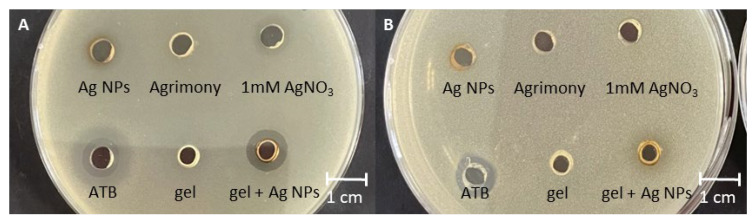
Antimicrobial activity of Ag NPs, *Agrimonia eupatoria* L., AgNO3, and in situ gel with and without nanoparticles against (**A**) *Escherichia coli* and (**B**) *Staphylococcus aureus*. ATB indicates the antibiotic gentamicin at a concentration of 50 μM/mL.

The silver nanoparticles prepared using plant extract were tested for antibacterial activity. To assess complex activity, two bacterial strains were exposed to the tested solutions. Escherichia coli and Staphylococcus aureus were used as representative Gram-negative and Gram-positive bacteria, respectively. The effectiveness of the silver nanoparticles was compared with that of the silver ions released from AgNO_3_ and an extract of *Agrimonia eupatoria* L. in the same concentration that was used for the biosynthesis. The antibacterial activity of an ISG with silver nanoparticles prepared with a plant extract was also tested. An ISG prepared with water without nanoparticles was used as a control. The results were compared with the effect of gentamicin at a concentration of 50 μg/mL (Figure 7).

The antibacterial activity of 1 mM of AgNO_3_ was tested to exclude its effect on bacteria. *E. coli* and *S. aureus* were resistant to 1 mM of AgNO_3_. The water extract from *Agrimonia eupatoria* L. showed no antibacterial activity against *E. coli* or *S. aureus*, as anticipated; therefore, agrimony can be used for wound healing thanks to the tannins being astringent and not because of antimicrobial activity. Our results agree with the work of Muruzovic, in which *E. coli*, *S. enterica*, and *S. typhimurium* showed resistance to water, acetone, diethylether, and ethanol extracts of *Agrimonia eupatoria* L. (>20 mg/mL). The water extract at a concentration of 2.5 mg·mL^−^^1^ had a minimal inhibitory concentration for *S. aureus* [60]. In our case, the concentration used (1 mg·mL^−^^1^) was below the MIC limit; therefore we did not observe any antimicrobial effect for the water extract. Watkins et al. showed the antimicrobial effects of the root; specifically, *A. eupatoria* inhibited the growth of *E. coli* by >60% at 200 μg/mL in water decoctions and 25% in ethanol extracts [61]. Some authors have observed the antibacterial activity of *Agrimonia eupatoria* L. extracts against *E. coli*, *S. aureus*, and other bacterial strains; however, the extracts were prepared differently, with much higher concentrations of plant material [62,63,64].

Silver in the form of nanoparticles showed no antibacterial activity. This was surprising, as many articles have described the opposite finding. Nanoparticles prepared via green synthesis using *Moringa oleifera* leaf at a concentration of 1 mM and incorporated into bacterial cellulose and filter paper discs exhibited antimicrobial activity against *Staphylococcus aureus* ATCC 6538 and *Pseudomonas aeruginosa* ATCC 9027 [65]. Silver nanoparticles synthesized with probiotic bacteria showed increased antibacterial activity and increased antioxidant activity [66]. The bactericidal potential of NPs depends on the shape, size, and concentration of the particles. Size has been shown to have a strong effect on activity. Spherical and rod-shaped nanoparticles showed lower biocidal activity against *E. coli* compared to truncated triangular silver nanoplates [67]. We synthesized spherical nanoparticles, which probably also affected the activity against *E. coli* and *S. aureus*. In our case, the Ag NPs were negatively charged (zeta potential around −30), so they were repelled to the negatively charged cell wall of the *E. coli*. A Ag NP concentration of 1 mM without antibacterial support from plant material is not sufficient to stop bacterial growth. It was shown in a previous study using different plant materials that silver nanoparticles prepared via green synthesis demonstrate antimicrobial activity, but the plant extract has no effect [68].

No activity was detected in the ISG without Ag NPs. This was expected since nothing in the chemical composition of the ISG had an effect against bacterial strains. Surprisingly, antibacterial activity was observed in the case of the ISG prepared with Ag NPs against *E. coli* but not against *S. aureus*. The activity against Gram-positive and Gram-negative strains varied due to the differing structural and molecular composition of the bacterial cell wall and the sensitivity to Ag NPs. From the above results, we can conclude that the antibacterial activity of the ISG with Ag NPs was based only on the presence of Ag NPs. It was evident that Ag NPs had better antimicrobial activity in the form of ISG than in a solution. Incorporation of Ag NPs into ISG changed the surface charge, as determined by the zeta potential measurement. The negatively charged *E. coli* bacteria were resistant to Ag NPs alone but, in combination with ISG, the activity was stronger than that of the antibiotic gentamicin (50 μM/mL). Negatively charged Ag NPs were repulsed by the negatively charged cell wall of *E. coli*. In contrast, Ag NPs in ISG with almost no charge had no electrochemical barrier and were, therefore, effective. The ISG also altered the distribution, aggregation, and release of silver ions from nanoparticles, which could have also contributed to the antibacterial activity.

## 4. Conclusions

Silver nanoparticles were synthesized using the medicinal plant *Agrimonia eupatoria* L. We prepared monodispersed Ag NPs with a spherical shape and a size of about 20 nm. It was found that the active organic substances from *Agrimonia eupatoria* L. were bound to the surface of the nanoparticles. The *Agrimonia eupatoria* L. suspension with silver nanoparticles was successfully used for the preparation of in situ gels. The antibacterial activities of the ISG were stronger that those of the silver nanoparticles, which showed no activity. Such in situ gels have great potential for use in wound healing. Due to their ability to change their liquid structure to that of a gel, they can stick to the mucous membrane more strongly and for a longer period of time. The positive effect was strengthened by the anti-inflammatory and antioxidant activities of the organic compounds of *Agrimonia eupatoria* L. Another positive effect was based on the antimicrobial activity of the silver nanoparticles. In conclusion, termosensitive in situ gels with silver nanoparticles prepared with plant extract can improve mucosal wound therapy.

## Figures and Tables

**Table 1:** Chromatographic data for compounds identified with HPLC in the aqueous extract of *Agrimonia eupatoria* L.

Compound	Rt (Min)	UV Max (nm)	Calibration Range (mg·L^−1^)	Regression			Concentration (mg·L^−1^)
				Slope	Offset	R^2^	
Gallic acid	3.87	214, 274	0.01–20	0.1740	−0.0603	0.9994	0.83
Chlorogenic acid	7.72	248, 328	0.01–20	0.1581	−0.0048	0.995	1.03
Caffeic acid	9.11	245, 326	0.01–20	0.2738	−0.0671	0.995	1.39
*p*-Coumaric acid	11.40	309	0.01–20	0.3341	−0.0603	0.9988	3.83
*o*-Coumaric acid	13.35	279, 327	0.01–20	0.1504	−0.0268	0.9987	3.06
Rutin trihydrate	14.23	260, 357	0.01–20	0.0722	−0.0221	0.9991	6.56
Rosmarinic acid	14.54	250, 331	0.01–20	0.1513	−0.0367	0.9991	N.D.
Cinnamic acid	15.39	280	0.01–20	0.4832	−0.0886	0.9986	0.33
Quercetin	18.60	259, 374	0.05–20	0.1454	−0.1157	0.9968	N.D.

N.D.—not detected.

**Table 2:** Assigned characteristic bands in the FTIR spectra (wavenumbers (cm^−1^)).

	ν(O-H)	ν(C-H)	ν(C-H)	ν(C=O)	ν(C=C) ν(C=O) δ(N-H)	δ(C-OH) Phenol	δ(CCH) δ(CH_3_) ν(−C−O−C) ν(C–O) Alcohol ν(C–N) ν(C–C)
*Agrimonia eupatoria* L.	3222	2916	2848		1602	1396	1246–1043
supernantant after AgNP synthesis	3222	2914	2846	1720	1604	1371	1236–1039
Ag NPs	3224	2914	2846		1637, 1514, 1456		

## Data Availability

Not applicable.
